# Self-nanoemulsifying drug delivery system (SNEDDS) mediated improved oral bioavailability of thymoquinone: optimization, characterization, pharmacokinetic, and hepatotoxicity studies

**DOI:** 10.1007/s13346-022-01193-8

**Published:** 2022-07-13

**Authors:** Charul Rathore, Chetna Hemrajani, Abhishek Kumar Sharma, Piyush Kumar Gupta, Niraj Kumar Jha, Alaa A. A. Aljabali, Gaurav Gupta, Sachin Kumar Singh, Jen-Chang Yang, Ram Prakash Dwivedi, Kamal Dua, Dinesh Kumar Chellappan, Poonam Negi, Murtaza M. Tambuwala

**Affiliations:** 1 University Institute of Pharma Sciences, Chandigarh University, Ajitgarh, Punjab India; 2 School of Pharmaceutical Sciences, Shoolini University of Biotechnology and Management Sciences, Solan, 173 212 India; 3 Department of Life Sciences, School of Basic Sciences and Research (SBSR), Sharda University, Knowledge Park III, Greater Noida, Uttar Pradesh 201310 India; 4 Department of Biotechnology, School of Engineering & Technology (SET), Sharda University, Greater Noida, Uttar Pradesh 201310 India; 5 Department of Pharmaceutics and Pharmaceutical Technology, Yarmouk University—Faculty of Pharmacy, Irbid, 21163 Jordan; 6 School of Pharmacy, Suresh Gyan Vihar University, Jagatpura, Jaipur 302017 India; 7 School of Pharmaceutical Sciences, Lovely Professional University, Phagwara, Punjab India; 8 Graduate Institute of Nanomedicine and Medical Engineering, College of Biomedical Engineering, Taipei Medical University, Taipei, 110-52 Taiwan; 9 School of Electrical and Computer Science Engineering, Shoolini University, Solan, (H.P.) India; 10 Discipline of Pharmacy, Graduate School of Health, University of Technology Sydney, Ultimo, NSW 2007 Australia; 11 Department of Life Sciences, School of Pharmacy, International Medical University, Bukit Jalil 57000, Kuala Lumpur, Malaysia; 12 School of Pharmacy and Pharmaceutical Sciences, Ulster University, Coleraine, County Londonderry, BT52 1SA Northern Ireland UK; 13 Lincoln School of Medicine, University of Lincoln, Brayford Pool Campus, Lincoln, LN6 7TS United Kingdom

**Keywords:** SNEDDS, Thermodynamic stability, Bioavailability, Hepato-toxicity, In vitro release kinetics

## Abstract

Thymoquinone (TQ) is an antioxidant, anti-inflammatory, and hepatoprotective compound obtained from the black seed oil of *Nigella sativa*. However, high hydrophobicity, instability at higher pH levels, photosensitivity, and low oral bioavailability hinder its delivery to the target tissues. A self-nanoemulsifying drug delivery system (SNEDDS) was fabricated using the microemulsification technique to address these issues. Its physicochemical properties, thermodynamic stability studies, drug release kinetics, in vivo pharmacokinetics, and hepatoprotective activity were evaluated. The droplet size was in the nano-range (< 90 nm). Zeta potential was measured to be −11.35 mV, signifying the high stability of the oil droplets. In vivo pharmacokinetic evaluation showed a fourfold increase in the bioavailability of TQ-SNEDDS over pure TQ. Furthermore, in a PCM-induced animal model, TQ-SNEDDS demonstrated significant (*p* < 0.05) hepatoprotective activity compared to pure TQ and silymarin. Reduction in liver biomarker enzymes and histopathological examinations of liver sections further supported the results. In this study, SNEDDS was demonstrated to be an improved oral delivery method for TQ, since it potentiates hepatotoxicity and enhances bioavailability.

**Graphical abstract:**

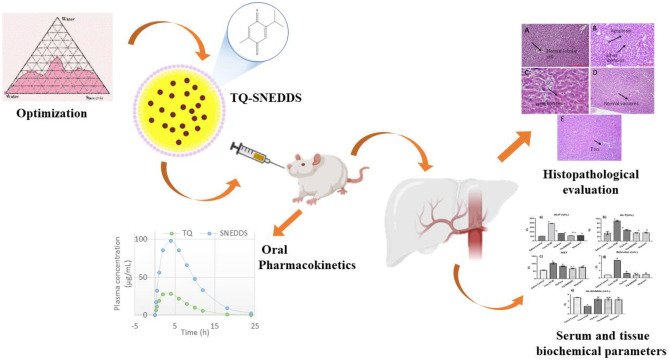

## Introduction

**Fig. 1:**
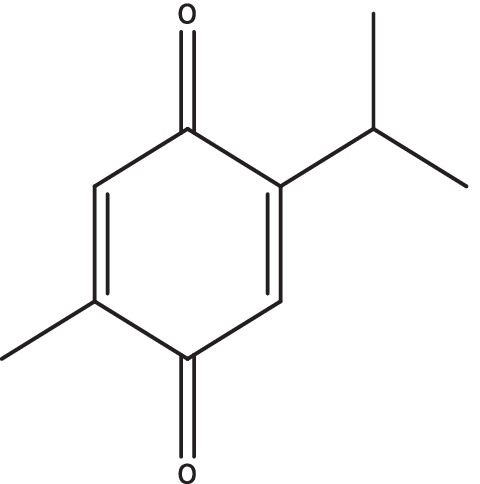
Molecular structure of thymoquinone

Thymoquinone (TQ) is the principal constituent in the seed oil of the plant *Nigella sativa* (Ranunculaceae) [1]. The biomolecule (Fig. 1) can significantly reduce the transactivation of hepatic stellate cells (HSCs) via its antioxidant, anti-inflammatory, anti-apoptotic, and radical-scavenging abilities and exert hepatoprotective effects [2–4]. TQ relieves liver oxidative stress by directly scavenging free radicals and/or indirectly by reactivating endogenous enzymatic and non-enzymatic antioxidants [5, 6]. Furthermore, TQ alleviates inflammation in liver tissues by inhibiting the cyclooxygenase, 5-lipoxygenase and hence eicosanoid production in leukocytes [7]. However, few limitations restrict its potential as an ideal drug candidate such as high hydrophobicity, pH instability, photo-sensitivity, high first-pass metabolism, and poor systemic bioavailability [8–11]. Bioactive molecules have increasingly been encapsulated in lipid nanocarriers for enhanced oral delivery as nanoscale formulations. These nanocarriers are capable of enhancing dissolution while at the same time protecting the drug from the harsh physiological environment [12]. In addition, they shield the drug from the body’s normal first-pass metabolism, thereby improving its overall efficacy [4, 13, 14]. Nanocarriers with rationally designed pharmacokinetic profiles may increase the therapeutic index of drugs [5]. They may also frequently allow for controlled and localized drug release, depending on the precise design of the therapy [15].

Amid various lipid-based nanocarrier systems, self-nanoemulsifying drug delivery systems (SNEDDSs) are reported to be superior, owing to their simple and easy preparation method, thermodynamic stability, and scalability. They are composed of lipids, surfactants, and cosurfactants and form mixed micelles nano-droplets of an emulsified lipid when exposed to a digestive motility environment of the gastrointestinal (GI) tract. When these nano-micelles enter the mucosal surface in the GI tract, they facilitate a relatively higher drug uptake vis-à-vis conventional drug solutions [3].

Phytochemicals previously formulated using SNEDDS for oral delivery include genkwanin [16], luteolin [17], silybin [18], diacerein [19], sesamin [20], 4-allypyrocatechol [21], bruceine D [22], epiisopiloturine [23], quercetin [24], isoliquiritigenin [25], tetrandrine [26], apigenin [27], myricetin [28], resveratrol [29], naringenin [30], silymarin [31], cannabidiol [32], liquiritin [33], glaucocalyxin [34], among others. Previously, we have shown that TQ encapsulation in the SLNs employing phospholipid as cosurfactant could improve its oral bioavailability [35]. Consequently, we have attempted to develop TQ-SNEDDS considering the benefits SNEDDS offers as a lipidic nanocarrier. We formulated TQ-loaded SNEDDS using the microemulsification technique with Labrafil M2125 as oil, Tween 80, Plurol oleique as surfactant/co-surfactant (S_mix_) mixture, and water. A mathematical model was used to investigate the mechanism of TQ release from SNEDDS formulation. Finally, the in vivo pharmacokinetics and hepatoprotective action of TQ-SNEDDS in Wistar rats were also evaluated.

## Materials and methods

### Materials

TQ was purchased from M/s Nice Chemicals Pvt. Ltd., Cochin, India. Other reagents, namely, sodium dihydrogen phosphate, potassium dihydrogen phosphate, and sodium hydroxide (NaOH), were made available from Sigma-Aldrich, Mumbai, India. Labrafil M 2125 CS, Labrafac Lipophile WL 1349, Labrafac PG, Labrafil, and Compritol 888 ATO were received ex gratia from M/s Gattefosse, Saint-Priest, France. M/s Merck Specialties Pvt. Ltd. India supplied ethanol (EtOH) and hydrochloric acid (HCl). Span 80 and Tween 80 were obtained from M/s Qualikem Fine Chemicals Pvt. Ltd. Vadodara, India. Glyceryl monostearate (GMS) and stearic acid were obtained from Hi-Media Pvt. Ltd., Mumbai, India.

A total of 32 albino Wistar rats weighing 250–300 g were used in this work. Rodents have been extensively used for studying liver disease as they are low cost, easy to procure, and predictable, and experience with this model has been widely substantiated. The animals were sourced from the National Institute of Pharmaceutical Education and Research, Mohali, Punjab, India. The animals were then placed in the animal house establishment of Shoolini University, Solan, HP, India, where we had conducted the experimental studies. The rats were housed in plastic bottom cages with an unrestricted availability of food and water. The temperature and relative humidity for animals were adjusted at 25 ± 2 °C and 45 ± 5%, respectively. Before actual testing, all animals were provided a 7-day acclimatization period at the experimental facility.

### Methods

#### Solubility studies

For the study, oil samples, namely, Labrafil M 2125 CS, Labrafac Lipophile WL 1349, and Labrafac PG, were screened based on TQ miscibility (Table 1). A surplus of TQ was added to the 10-mL vehicle and vortexed for 5 min to attain a homogenous mixture [36]. In the next step, the mixture was incubated in a shaking water bath regulated thermostatically (Remi, Mumbai, India) at a controlled temperature of 30 ± 1 °C for 72 h to achieve equilibrium. Afterward, the mixture was then centrifuged at 3000 rpm for 10 min, and the weighed quantity of the resulting supernatant was dissolved with ethanol and analyzed with HPLC equipment after suitable dilution [37].

**Table 1:** Solubility of TQ in various oils

Oil concentration (%)	Miscibility	Clarity
Labrafil® M 2125	+ + +	Very clear
Labrafac® PG	+ +	Slightly hazy
Labrafac Lipophile® WL 1349	+	Hazy

#### Pseudo-ternary phase diagram fabrication

Pseudo-ternary phase diagrams were created using the traditional titration method to obtain a clear self-emulsifying region [37]. All components, namely, oil, water, and surfactant–co-surfactant mixture (S_mix_), were mixed to get an optimum concentration. S_mix_ in the different ratios (1:1, 2:1, and 3:1) was dispersed in oil in the ratios of 1:9, 2:8, 3:7, 4:6, 5:5, 6:4, 7:3, 8:2, and 9:1 and titrated with water. Vortexing was used to make the mixture uniform, and it was visually checked for signs of turbidity.

The end-point was determined to be the point at which turbidity appeared, and the amount of water added was calculated on a weight-by-weight basis [38]. The ternary phase diagram was plotted using software PCP Disso v2.08, Pune, India, and a point in the clear micro-emulsion region was selected as the appropriate point.

#### Preparation of SNEDDS

**Fig. 2:**
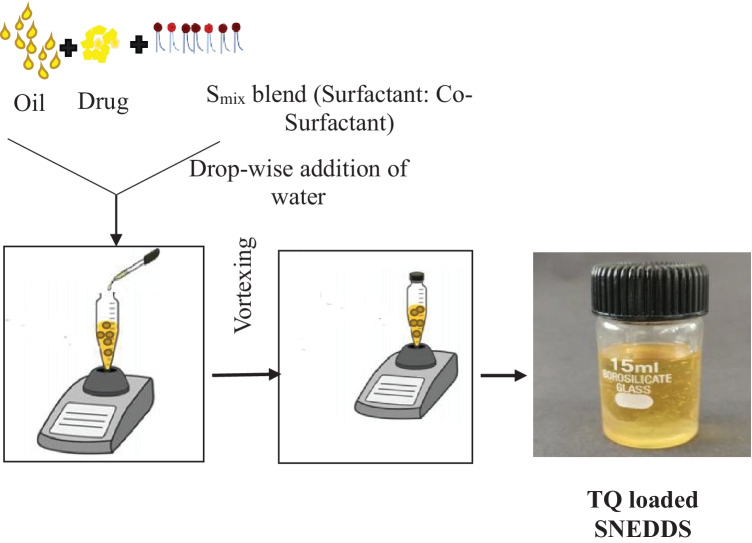
Schematic representation for SNEDDS preparation: isotropic mixture of TQ in oil phase, surfactants, and cosurfactants, resulting in extremely fine oil/water emulsions upon water addition

The nanoemulsion region of the pseudoternary phase diagrams was chosen for various compositions, and SNEDDS was made from them. First, a weighed amount of oil was taken, and the drug was dissolved in it by vortex mixing. Then, S_mix_ was added into the above mixture and stirred continuously to form a homogeneous mixture. Nanoemulsion was developed by continuously adding water drop by drop and stirring continuously. The method of preparing SNEDDS is depicted schematically in Fig. 2.

To create solid SNEDDS, we used an inert solid carrier like avicel because of its high surface area and excellent adsorption ability. Avicel powder and SNEDDS formulation (1 g) were mixed using a glass mortar to create a non-sticky solid powder. After that, the powder was passed through sieve no. 60 and kept at room temperature in a desiccator until further testing [39].

#### Thermodynamic stability studies for SNEDDS preparations

Various thermodynamic stability studies were carried out to check for any signs of instability among the various SNEDDS formulations prepared. These studies were performed by the method used and reported previously in the literature [36, 40].

##### Centrifugation studies

In the centrifugation study, all the formulated preparations were centrifuged for 30 min at 5000 rpm (REMI, India). A visual examination was performed to detect instabilities, such as phase separation, cracking, or creaming. Further testing was carried out on the stable formulations [36].

##### Self-emulsifying test

The self-emulsifying efficiency of SNEDDS was performed in a USP apparatus II. All the formulations (500 µL) were diluted with up to 500 mL (distilled water) and gently agitated at 50 rpm at 37 ± 0.5 °C. On the basis of the emulsification time, formulations were graded on a scale of A, B, and C [17].

Grade A: A clear nanoemulsion that forms quickly (in 1 min).

Grade B: This emulsion forms quickly and is less clear with a bluish-white appearance.

Grade C: Development of a fine milky emulsion within 2 min.

##### Heating and cooling cycle

The formulations were subjected to six heating and cooling cycles at 4 to 40 °C for at least 48 h. The formulations were analyzed for phase separation, creaming, and cracking [36].

##### Freeze thawing

Freezing thawing was carried out in three cycles at a temperature between − 21 and 25 °C for about 48 h. Following centrifugation for 5–10 min at 5000 rpm, all formulations were examined for instability like phase separation, creaming, and cracking [36, 40].

#### Transmittance studies

Transmittance of various SNEDDS formulations was observed spectrophotometrically at a wavelength of 254 nm by diluting 0.1 mL of the suspension up to 10 mL with distilled water against water as the blank (set at 100% transmittance) [9, 41, 42].

#### Globule size and zeta potential

Zetasizer 2000 HS (M/s Malvern Instruments Limited, Malvern, UK) with dynamic light scattering was used to determine globule size at ambient temperature. The experiment was conducted at the IIT Mandi (Himachal Pradesh), India. Detection angles were set at 90° for all measurements [43]. In distilled water, 0.1 mL of SNEDDS formulations was diluted to 100 mL and thoroughly mixed before being analyzed at room temperature. Following that, fluctuations in laser beam intensity were observed due to the Brownian motion of the particles after 1 mL of the test sample was placed in the cuvette [44].

#### Electron microscopy

The morphology of the SNEDDS was investigated by employing a transmission electron microscope (TEM). To accomplish this, a transmission electron microscope set up at the Indian Institute of Technology (IIT), Mandi (Himachal Pradesh), India, was used. In a 1:10 ratio, the SNEDDS formulation was diluted using distilled water [45]. Approximately 1–2 drops of diluted SNEDDS were placed on the carbon grid and examined at appropriate magnifications under the TEM [38].

The structure of the developed nanocarrier was also probed using a field emission-scanning electron microscope (FE-SEM) for the solid-SNEDDS. The samples were placed inside the vacuum chamber after being wrapped in carbon conductive tape. FE-SEM (Hitachi s-4800) installed at SAIF, Punjab University, Chandigarh, India, allowed moving electrons from the tungsten filament to interact with the sample, and the surface morphology of solid-SNEDDS was determined [46].

#### X-ray diffraction (XRD)

A typical XRD pattern was obtained at room temperature using copper Kα radiation (1.54060 A) at 45 kV and 40 mA via the X’Pert PRO diffractometer system (Panalytical, Netherlands) for pure TQ, solid-TQ-SNEDDS, and avicel. The analyses of each sample were conducted at a scan speed of 2 min^−1^ using an aluminum sample container continuously scanning between 5 and 40° in 2θ at 2 min^−1^ speed [47].

#### Diffraction scanning calorimetry (DSC)

The DSC patterns of pure TQ, solid TQ-SNEDDS, and avicel were measured using the NETZSCH leading thermal analysis (DSC204, F1 Phoenix) (Germany). On an aluminum plate, the samples were heated at a 10 °C/min rate between temperatures 20 and 350 °C. A 20-mL/min flow rate of inert argon was used as an effluent gas at a 20-mL/min flow rate to assess stability and compatibility [48].

#### In vitro drug release kinetics

TQ suspension (20 mg/kg distributed in 4%, 1 mL sodium carboxymethylcellulose (CMC-Na)) and TQ-SNEDDS suspensions were dispersed for 2 h in 0.1 N HCl (pH 1.2) and then for 12 h in phosphate buffer (pH 6.8) containing 2.5% (w/v) Tween 80 before being placed separately in MWC 1 kD dialysis bags. After placing the dialysis bags in 25 mL of release media, water bath shaker was maintained at 37 °C and 100 strokes/min [49]. Approximately 2 mL of diffusion medium was withdrawn at predetermined time intervals (0, 0.25, 0.5, 1, 2, 4, 6, 8, 10, and 12 h) and replenished to preserve the sink condition. To measure the cumulative release of TQ from SNEDDS formulations, HPLC was employed at 254 nm (λ_max_) following appropriate dilution(s) [37]. All measurements were done in triplicate. Mechanism of the drug release from SNEDDS was also carried out by fitting various mathematical models into in vitro release data, including Zero-order, First order, Higuchi model, and Korsermeyer Peppas model [29]. The kinetic model was chosen based on the regression coefficient (*r*^2^) with the highest value.

#### Oral pharmacokinetic determinations

In vivo pharmacokinetic measurements were conducted on a set of healthy albino Wistar rats. A standard laboratory diet and water were available to rats in standard conditions under room temperature and relative humidity of 55 ± 5%. There were two groups (*n* = 6) of six rats each. Due to the low aqueous solubility, Group I received TQ (20 mg/kg) distributed in 4% sodium carboxymethylcellulose (CMC-Na) (1 mL) orally. Group II received a single dose of TQ-loaded SNEDDS (20 mg/kg) each containing TQ equivalent to 20 mg (i.e., 2 mL). Under light anesthesia (ether), an estimated 0.5 mL of blood was drawn from the retro-orbital plexus at specified times (pre-dose, 1, 2, 4, 6, 8, 10, 12, 18, and 24 h) and placed into microcentrifuge tubes heparinized for analysis. Following this, the blood sample was centrifuged at 3000 rpm for 15 min. Centrifugation separated plasma, which was stored at −40 °C pending analysis. In a microcentrifuge tube, 200 µL of a plasma-containing drug (TQ) was added and precipitated with the same volume of acetonitrile (200 µL) [50]. Furthermore, to make the volume 1 mL, methanol was used. After that, the mixture was centrifuged at 6000 rpm for 10 min. The column was then injected with 20 µL of supernatant for the TQ analysis using HPLC [37]. The pharmacokinetic analysis was performed using MS Excel software to assimilate the results obtained into a one-compartment open body extravascular model and compute pharmacokinetic parameters C_max_, t_1/2_, t_max_, V_d_, AUC_(0–24)_, AUC_(0-∞)_, and F.

#### Hepatic toxicity triggered by PCM

Rats were split into five groups at random, containing four animals (*n* = 4). Normal saline was administered to group I (control), PCM p.o (650 mg/kg) was administered to group II (toxic control), Group III received TQ-suspension group (20 mg/kg), Group IV received TQ-loaded SNEDDS (20 mg/kg), and Group V was given silymarin tablet (SILYBON®) suspension (40 mg/kg) for 7 days. All groups except Group I (control) received PCM 650 mg/kg concurrently for 7 days, while normal control group rats were given only the vehicle (normal saline) [51]. A blood sample of about 0.5 mL was taken after 7 days under mild ether anesthesia from the retro-orbital plexus. Serum was separated from the blood by centrifuging at 1500 rpm for 15 min and frozen in non-heparinized tubes at 80 °C until further testing. Alanine aminotransferase (ALT), aspartate aminotransferase (AST), alkaline phosphate (ALP), bilirubin, and albumin were measured in the serum obtained [52]. On the last day of the study, cervical dislocation was used to sacrifice rats, and liver tissues were removed for histopathological evaluation.

#### Histopathological examination

After washing with phosphate buffer saline, the liver tissues were dried on tissue paper then stored in formalin solution 10%. A light microscope was used to observe the photographs after staining them with hematoxylin and eosin (H&E) to detect necrosis, hemorrhage, and inflammatory cells.

## Results and discussion

### Solubility study

Oil phase is an essential requirement for the preparation of drug-loaded SNEDDS. Hence, to construct the TQ-loaded SNEDDS, its solubility in different oils was assessed visually in terms of clarity. TQ was maximum soluble in Labrafil® M 2125 followed by Labrafac® PG and Labrafac Lipophile® WL 1349 as depicted in Table 1. For high drug-loading, solubility of drug in the oil is very important. Thus, considering the maximum solubility of TQ in Labrafil® M 2125, it was selected as oil phase for the construction of SNEDDS. Therefore, Labrafil® M 2125 was selected as oil phase for further preparation of SNEDDS. The maximum solubility of TQ in Labrafil® M 2125 could be because of the presence of constituents, which might have aided in the solubilization of the drug.

### Formulating SNEDDS

**Fig. 3:**
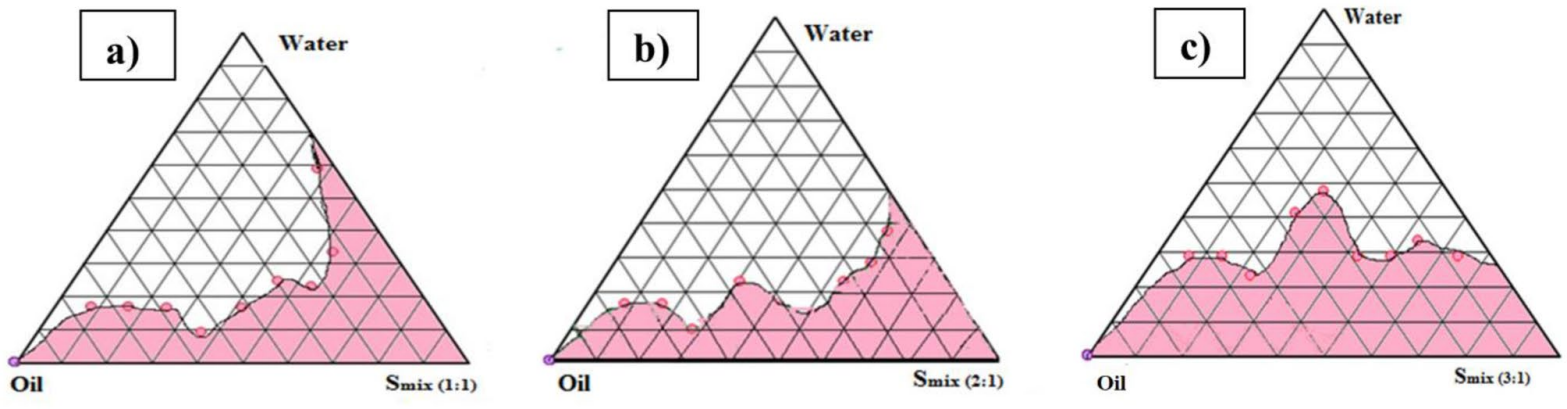
Pseudo-ternary phase diagrams of SNEDDS composed of oil (Labrafil M2125), S_mix_ (Tween 80: Plurol oleique), and water at various S_mix_ ratios (**a**) 1:1, (**b**) 2:1, and (**c**) 3:1

Using pseudoternary phase diagrams, the nanoemulsion regions were mapped, and appropriate concentrations of three components that could result in the formation of SNEDDS were determined (Labrafil® M 2125, Tween 80, and Plurol oleique®). Tween 80 and Plurol oleique® as S_mix_ were employed in varying ratios, namely, 1:1, 2:1, and 3:1, to construct three ternary phase diagrams. The ternary phase diagram, i.e., Fig. 3a where S_mix_ is 1:1, showed a small nanoemulsion zone, while Fig. 3b (S_mix_ 2:1) and Fig. 3c (S_mix_ 3:1) showed a significantly better nanoemulsion zone. This could be attributed to the high Tween 80 in S_mix_ ratio 3:1 vis-à-vis other ratio (2:1 and 1:1). Tween 80 is a surfactant with a high HLB value; thus, its high content in the 3:1 ratio could emulsify oil better at the oil–water interface. Co-surfactant (Plurol oleique) will be beneficial to form microemulsion at a proper concentration range. Tween 80 and Plurol oleique® in the ratio of 3:1 ratio were able to result in better emulsification at the oil–water interface, resulting in a bigger nanoemulsion region [53, 54]. However, an excessive amount of the co-surfactant will cause the system to become less stable for its intrinsic high aqueous solubility and lead to the droplet size increasing as a result of the expanding interfacial film [55]. From these three constructed ternary phase diagrams, a total of nine formulations were prepared (Table 2), loading drug at a fixed amount of oil, S_mix_, and water, for further analysis [56, 57].

**Table 2:** Composition and qualitative results of various SNEDDS formulations selected from pseudoternary phase diagrams

SNEDDS								
Code	**Oil (%)**	**S**_**mix**_** (%)**	**Water (%)**	**Centrifugation**	**Self-emulsification**	**Heating–cooling and freeze–thaw**	**Globule size (nm)**	**%Transmittance**
**S**_**mix**_** (1:1)**								
**F1**	20 (4)	50 (10)	30 (6)	Pass	C	Pass	200 ± 2.52	90.7 ± 0.23
**F2**	25 (5)	50 (10)	25 (5)	Fail	C	Fail	190 ± 1.53	92.2 ± 0.13
**F3**	30 (6)	45 (9)	25 (5)	Pass	B	Pass	150 ± 3.06	93.1 ± 0.19
**S**_**mix**_** (2:1)**								
**F4**	10 (2)	50 (10)	40 (8)	Pass	B	Pass	200 ± 5.13	91.6 ± 0.25
**F5**	30 (6)	50 (10)	20 (4)	Pass	C	Pass	200 ± 2	93.4 ± 0.30
**F6**	20 (4)	50 (10)	30 (6)	Pass	A	Pass	110 ± 3.21	94.7 ± 0.21
**S**_**mix**_** (3:1)**								
**F7**	20 (4)	50 (10)	30 (6)	Fail	B	Fail	300 ± 5.29	87.8 ± 0.20
**F8**	25 (5)	50 (10)	25 (5)	Pass	B	Pass	150 ± 2.65	95.3 ± 023
**F9**	30 (6)	50 (10)	20 (4)	Pass	A	Pass	90 ± 2.65	97.6 ± 0.13

Whereas *A* clear, *B* translucent, and *C* milky. All formulations included 200 mg of drug

Table 2 summarizes the qualitative findings of thermodynamic stability tests. Thermodynamic stability studies of nanoemulsions are important that distinguish nanoemulsions from emulsions. Nanoemulsions are more stable than emulsions and exhibit no phase separation, turbidity, creaming, or cracking on prolonged storage. When centrifugation, heating, and cooling cycles and freeze–thaw pump cycles were performed on almost all formulations (F1-F9), thermodynamic stability was established for them. Furthermore, all the formulations were subjected to self-emulsification tests and categorized into three grades, i.e., grade A, grade B, and grade C, based on emulsification time. It has been noted that most of the TQ-loaded SNEDDS formulations passed this test with grade A and grade B when diluted with the dissolution medium. As demonstrated by Senapati et al., this test is considered to have the best grade of A [36]*.* Furthermore, by using a thermodynamic stability study, one can assess every possibility of whether the formulations undergo precipitation or phase separation when exposed to gastrointestinal fluids.

### Characterization of SNEDDS

#### Globule size, PDI, and zeta potential

**Fig. 4:**
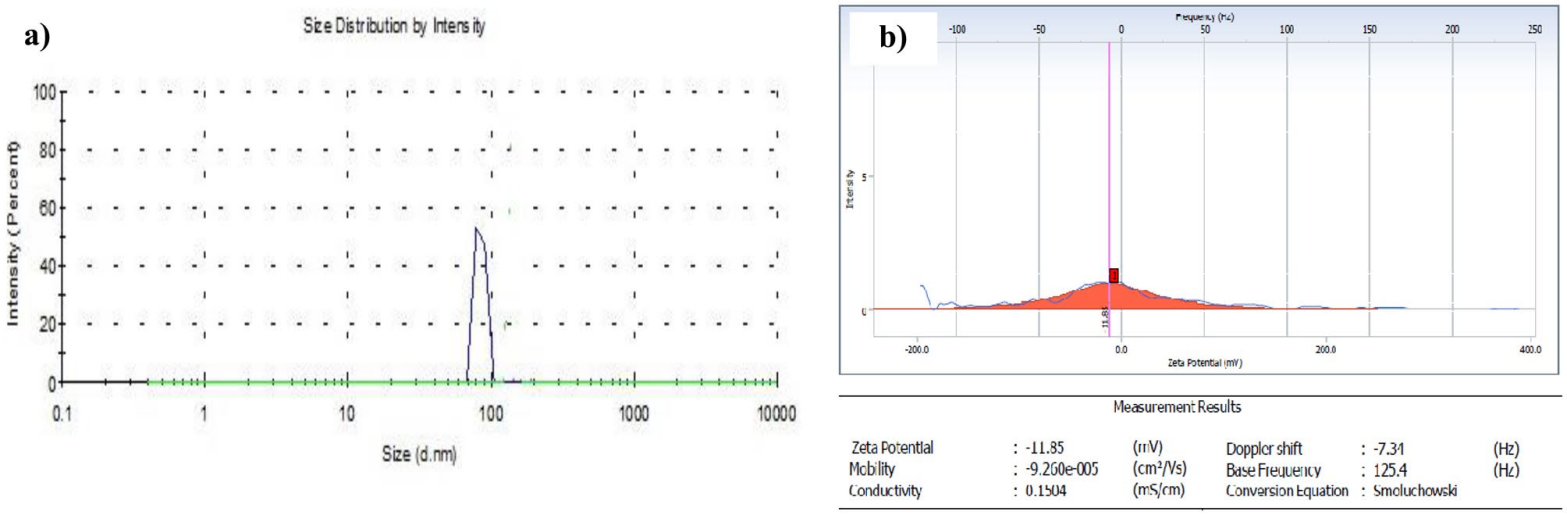
Characterization of optimized TQ-loaded SNEDDS. (**a**) Particle size distribution graph, (**b**) Zeta potential

The globule sizes of various SNEDDS formulations are listed in Table 2. The sizes of the globules ranged between 90 and 300 nm. The SNEDDS formulation chosen from the pseudoternary phase diagram (Fig. 3c) having S_mix_ ratio 3:1 revealed the smallest size. A greater concentration of Tween 80 having strong emulsifying capacity at the oil/water interface may explain homogenous nano-size droplets of the formulation. Furthermore, the high surfactant concentration led to decreased interfacial tension at the oil/water interface, which favors the construction of nanoemulsions with smaller droplets [57]. Based upon the thermodynamic stability studies (centrifugation, self-emulsification, heating–cooling, and freeze-thawing) and globule size, formulation F9 was selected, which has an oil phase (30%), S_mix_ (50%), and water (20%), respectively (Table 2), for further evaluation. The PDI of the optimized formulations was found to be 0.312. The low PDI value indicated the uniformity and narrow size distribution of the polydispersed phase. The zeta potential of TQ-SNEDDS was found to be negative (−11.35 mV), as shown in Fig. 4. Plurol oleique contains fatty acid esters, which account for the overall negative charge on TQ-SNEDDS. Because of the high energy barrier between particles, negative zeta potential value indicates excellent colloidal stability [58].

#### Percentage transmittance

Percentage transmittance of the total nine formulations was studied. It was observed that increase in surfactant ratio increased the percentage transmittance, indicating the transparent behavior of the formulations. Percentage transmittance value closer to 100% gave an idea of droplet size in nanometer range. The droplet size of the emulsion is a crucial factor in self-emulsification performance because it determines the rate and extent of drug release as well as absorption [59].

#### Morphology

**Fig. 5:**
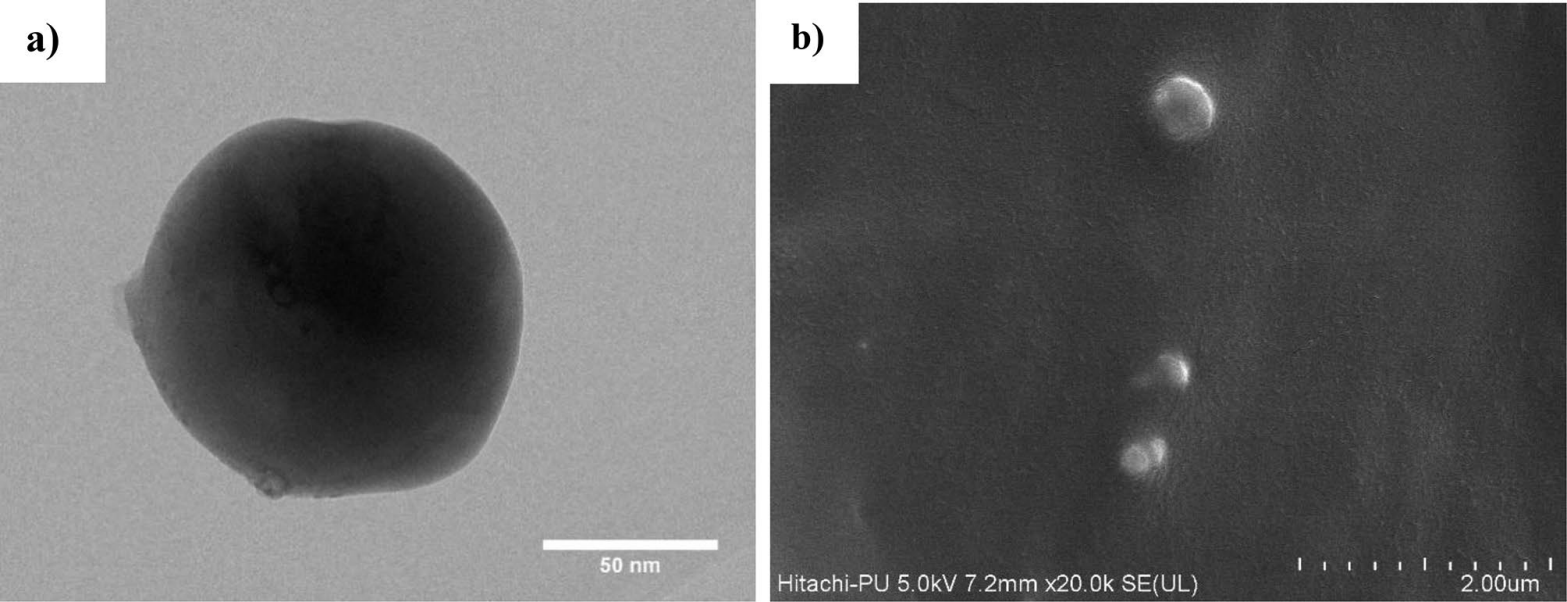
Morphology of TQ-SNEDDS observed by (**a**) transmission electron microscopy (at 60,000 ×) and (**b**) scanning electron microscopy (at 30,000 ×)

The morphology of SNEDDS was visualized by employing TEM and FE-SEM, as shown in Fig. 5a. SNEDDS appeared to be spherical as viewed through a TEM and agreed with the findings of the globule size obtained employing the DLS. In the FE-SEM images, the preparations of SNEDDS also appeared nearly spherical. Crystalline drug particles could not be observed, which confirms TQ entrapment inside SNEDDS globules, as shown in Fig. 5b.

#### XRD

**Fig. 6:**
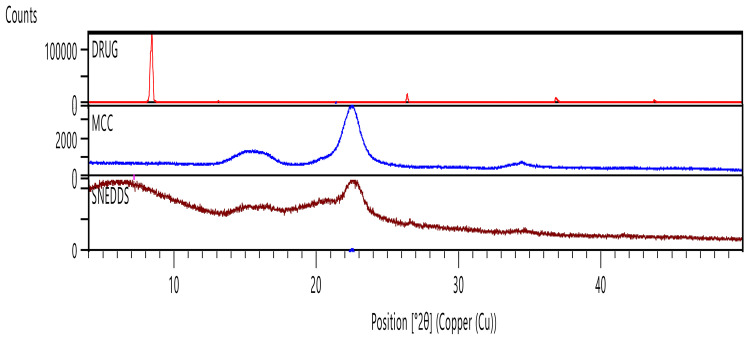
XRD pattern of TQ, avicel, and SNEDDS

The XRD profiles of TQ, avicel, and SNEDDS are displayed in Fig. 6. According to the XRD pattern of the TQ, it has high crystallinity. On the other hand, the intrinsic crystalline peak of TQ is absent in TQ-loaded SNEDDS, which means complete solubility and stability of the TQ inside the SNEDDS. This indicates that the drug was molecularly dispersed in the SNEDDS matrix [60].

#### DSC

**Fig. 7:**
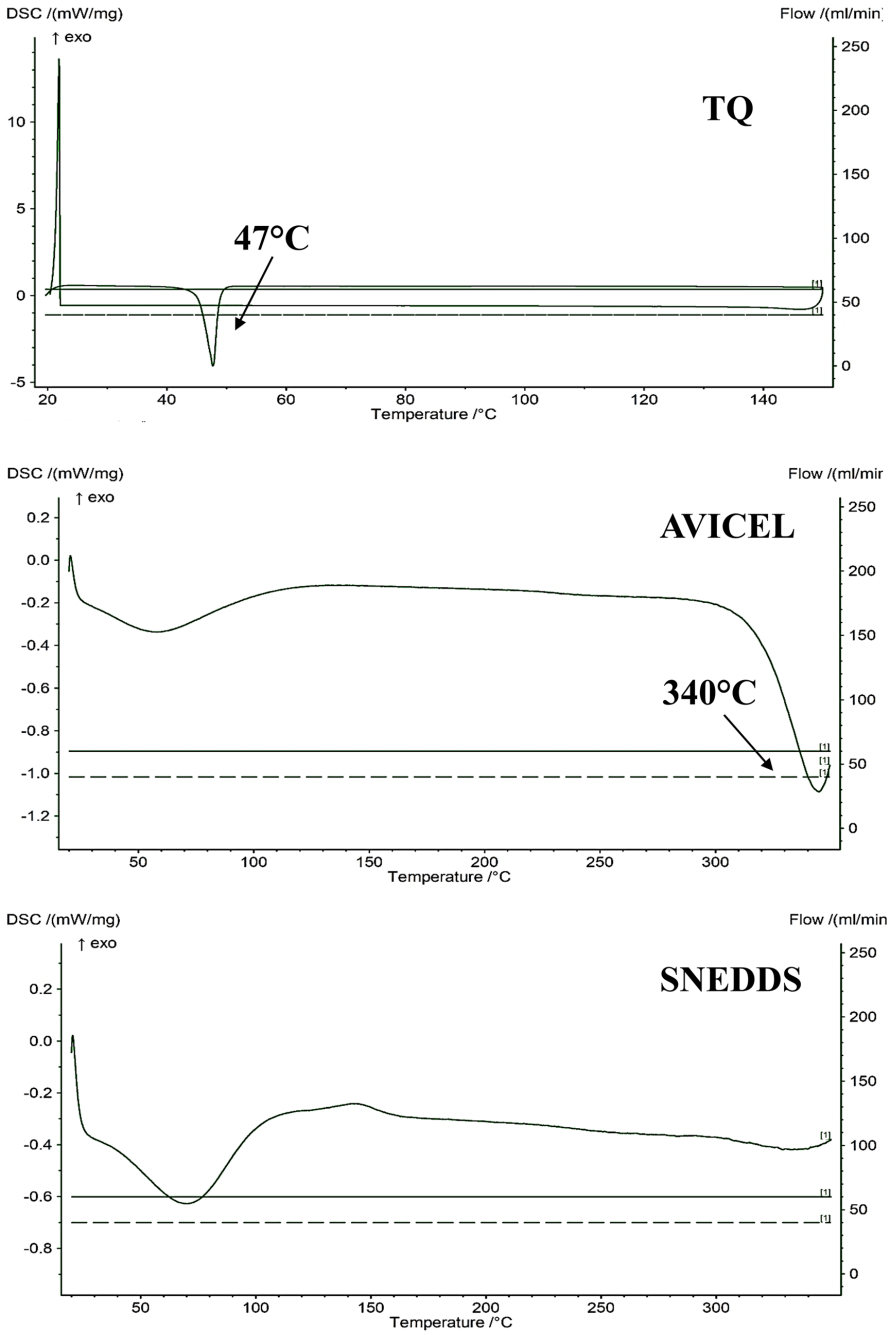
DSC thermogram of (**a**) TQ, (**b**) avicel, and (**c**) SNEDDS

The melting points of TQ, avicel, and SNEDDS were discovered using DSC analysis (Fig. 7). The TQ thermogram showed a pronounced endothermic peak around 47 °C, consistent with the literature-stated melting point range (45–50 °C). However, avicel exhibited a sharp endothermal peak around 340 °C, as shown in Fig. 7. The DSC thermogram of TQ-SNEDDS did not show any peak of TQ. This indicates the complete entrapment and solubilization of TQ in the SNEDDS formulation. Furthermore, it also suggests the transition of the physical state of the drug from crystalline to amorphous, and thus the transitions of TQ into high-energy form with a high disorder may favor its enhanced solubility [58].

#### In vitro release kinetics

**Fig. 8:**
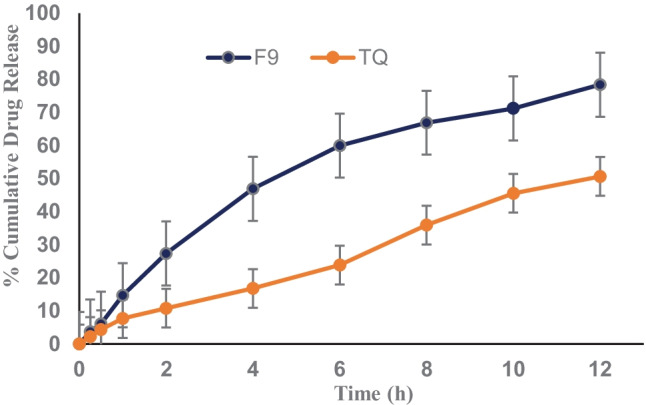
In vitro cumulative drug release profile (%) of (**a**) optimized TQ-SNEDDS (F9) and (**b**) pure TQ

In vitro release profiles of SNEDDS and pure TQ are shown in Fig. 8. In this study, the sink conditions were maintained by adding 2.5% (w/v) Tween 80 in the receptor medium. A substantial amount of the drug (nearly 80%) was released from the SNEDDS for up to 12 h compared to only 50% for plain TQ. The high and sustained release of the drug could be attributed to the nanosized droplets, composition, and better solubilization of SNEDDS in the release medium. First, small globule sizes in TQ-SNEDDS formulations allowed for a large surface area for drug release, thus enabling a faster release rate. Second, the presence of S_mix_ in the SNEDDS formulation improved TQ wetting of TQ and facilitated drug release [61, 62]. Finally, as the oil–water interface at the interface of nanoemulsions has low surface energy, the solubilization of drugs in the release medium occurs immediately due to spontaneous formation of nanoemulsion [63].

**Fig. 9:**
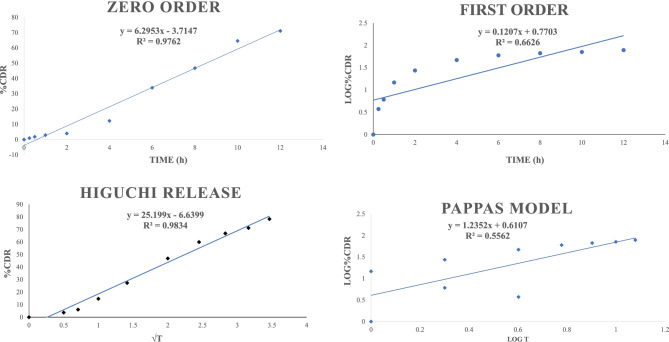
In vitro release kinetic studies of optimized TQ-loaded SNEDDS formulation for the computation of *r*.^2^ value using (**a**) Zero-order, (**b**) First order, (**c**) Higuchi matrix, and (**d**) Kosermeyer–Peppas model

TQ was released in a controlled pattern from SNEDDS and best fitted with Higuchi release kinetics with the highest *r*^2^ value (0.9834) (Fig. 9), indicating the drug release by diffusion in a slow and sustained manner.

### In vivo pharmacokinetic studies of SNEDDS

**Fig. 10:**
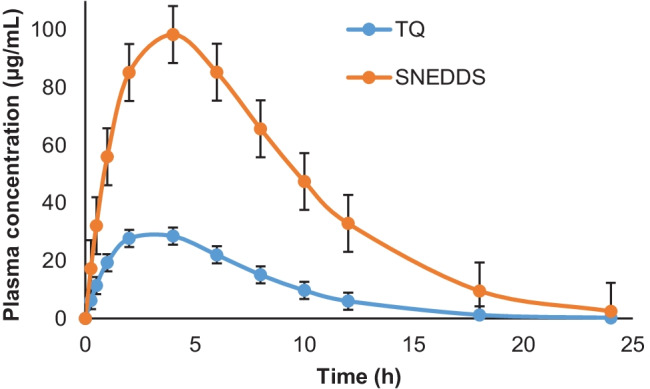
Comparative plasma concentration vs. time profiles of TQ and TQ-SNEDDS

The plasma concentration–time profile graph of TQ suspension and TQ-SNEDDS is depicted in Fig. 10 and their corresponding pharmacokinetic data are shown in Table 3. Figure 10 shows that TQ-SNEDDS significantly improved TQ absorption in vivo compared to TQ suspension. AUC_(0-t)_ and AUC_(0-∞)_ for TQ-loaded SNEDDS were 970.34 and 980.73, respectively. The C_max_ value was found to be 98.92 µg/mL. Furthermore, the oral bioavailability of TQ-loaded SNEDDS was four times higher than that of the TQ suspension. This improved oral bioavailability of the TQ from the SNEDDS matrix could be due to several possible mechanisms. Firstly, owing to the nanoscale size, the surface area is significantly increased which enhances the adhesion of nanocarriers to M-cells of the Peyer’s patches and gastric residence time, which further enhances the close contact of TQ-loaded SNEDDS formulation to the epithelial membrane and thus enhances oral absorption. Secondly, TQ-loaded SNEDDS may enhance the lymphatic transport of drugs by producing chylomicrons from the enterocytes. Furthermore, chylomicrons transport the drugs into lymphatic vessels, thus guarding the drug against hepatic first-pass metabolism and enhancing the oral bioavailability [64, 65]. Thirdly, drug-loaded lipidic nanocarriers come in contact with GI fluids, and mixed micelles are formed as a result and facilitate the oral absorption of hydrophobic drugs by the action of gastric lipase enzyme [66].

**Table 3:** Pharmacokinetic parameters of TQ-SNEDDS and suspension of TQ (mean SD) in rats (*n* = 6) after oral administration

Pharmacokinetic parameters	TQ suspension	TQ-loaded SNEDDS
C_max_ (µg/mL)	28.34	98.92
t_1/2_ (h)	2.0	2.63
t_max_ (h)	3.0	3.6
Ke	0.32	0.26
V_d_ (L/kg)	0.23	0.02
CL (L//kg/h)	0.07	0.03
AUC (0–24) (µg.h/mL)	242.82	970.34
AUC (0-inf) (µg.h/mL)	241.22	980.73
Relative bioavailability (F)	-	401.3%

### Serum biochemical estimation

**Fig. 11:**
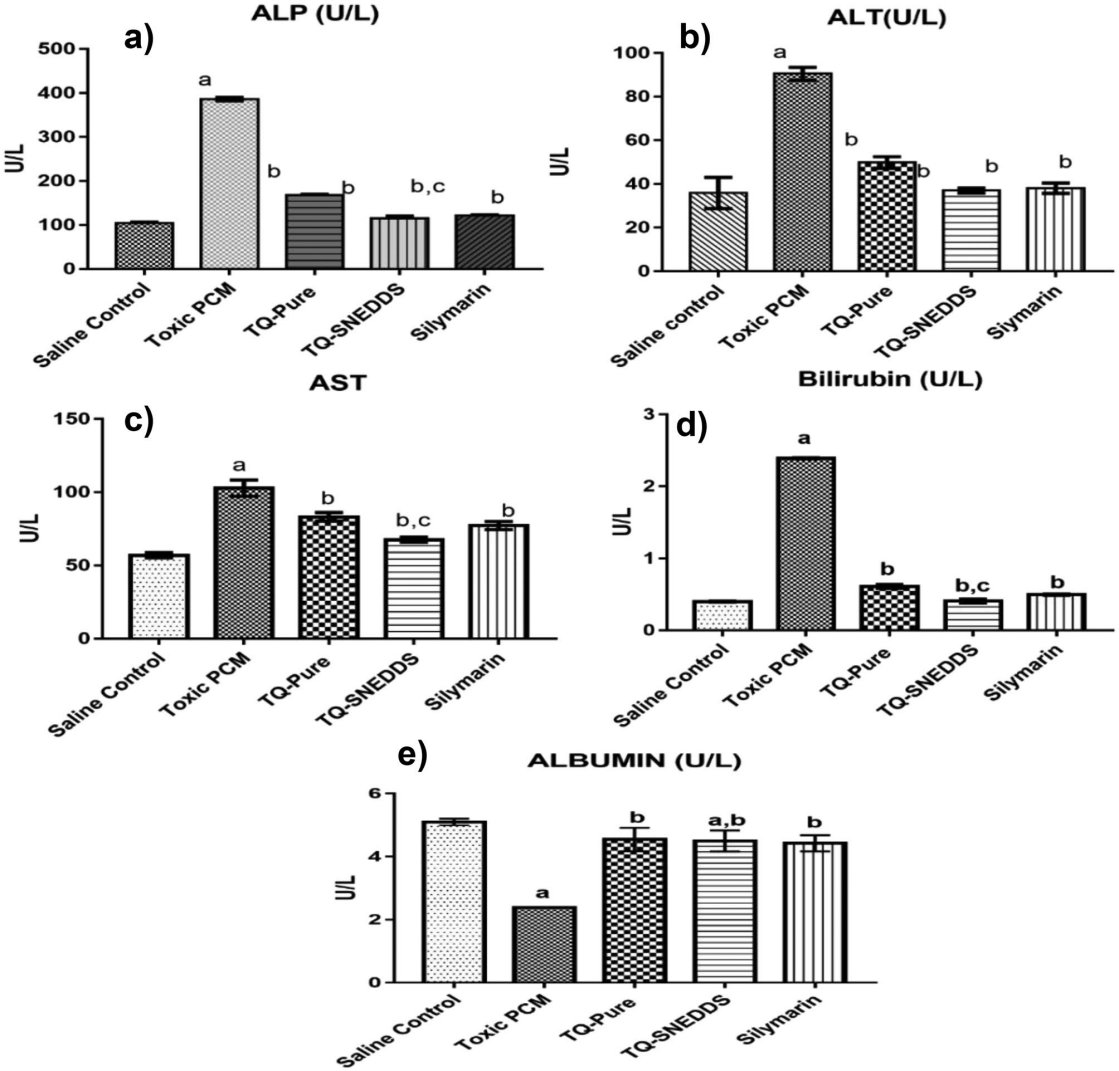
The impact of different formulations on serum and tissue biochemical parameters. Data is presented as mean ± SD (*n* = 4) and analyzed by one-way analysis of variance followed by Tukey’s multiple comparison test. a^*P*^ < 0.001 versus saline control, b^*P*^ < 0.001 vs. toxic PCM, and c.^*P*^ < 0.05 vs. silymarin

In Wistar rats, the in vivo hepatoprotective effects of TQ suspension, TQ-SNEDDS, and a standard drug (silymarin) against PCM-induced hepatotoxicity were investigated. Hepatotoxicity was reported after PCM administration. Table 4 and Fig. 11 summarize the results of various biochemical parameters (ALT, AST, ALP, albumin, and total bilirubin) for different groups. A sharp increase in marker enzymes indicated severe liver damage in the toxic group. PCM administration resulted in a significant increase in ALP, ALT, AST, bilirubin, and albumin levels (*p* < 0.001) in all other groups compared to the control group. After 7 days of treatment, all of the treated groups had lower levels of ALP, ALT, AST, bilirubin, and albumin. Silymarin and plain TQ suspension showed nearly identical levels of biomarker enzymes.

**Table 4:** Serum and tissue biochemical parameters after optimized TQ-PNC and TQ treatments (mean ± SD, *n* = 4)

Groups	ALT (U/L)	ALP (U/L)	AST (U/L)	Bilirubin (mg/dl)	Albumin
Saline control	36 ± 10.39	103.98 ± 2.52	58.8 ± 3.11	0.39 ± 0.02	4.8 ± 0.08
Toxic control (PCM)	86.8 ± 6.51	387.1 ± 5.43	101.93 ± 5.64	2.37 ± 0.02	3.3 ± 0.06
TQ suspension	46.39 ± 7.58	168.38 ± 1.99	78.96 ± 4.55	0.59 ± 0.04	4.7 ± 0.22
TQ-SNEDDS	37.1 ± 2.60	120.4 ± 5.20	70.8 ± 4.90	0.4 ± 0.02	5.1 ± 0.28
Silymarin	44.9 ± 6.01	122.11 ± 1.53	72.87 ± 3.44	0.51 ± 0.02	4.2 ± 0.11

The results were based on the averages of three replicate samples. Data is presented as mean ± SD (*n* = 3) (*p* < 0.001)

On the other hand, TQ encapsulation inside the SNEDDS matrix helped to significantly reduce liver biomarker enzymes (*p* < 0.05). This significant improvement in TQ-SNEDDS hepatoprotective performance can be attributed to nanocarriers smaller than 100 nm passing through endothelial fenestrations and effectively targeting HSCs (in rats). Moreover, a lipidic carrier also simplifies the delivery of the drug directly into the lymphatic system via the M cells in Payer’s patches.

### Histopathological evaluation

**Fig. 12:**
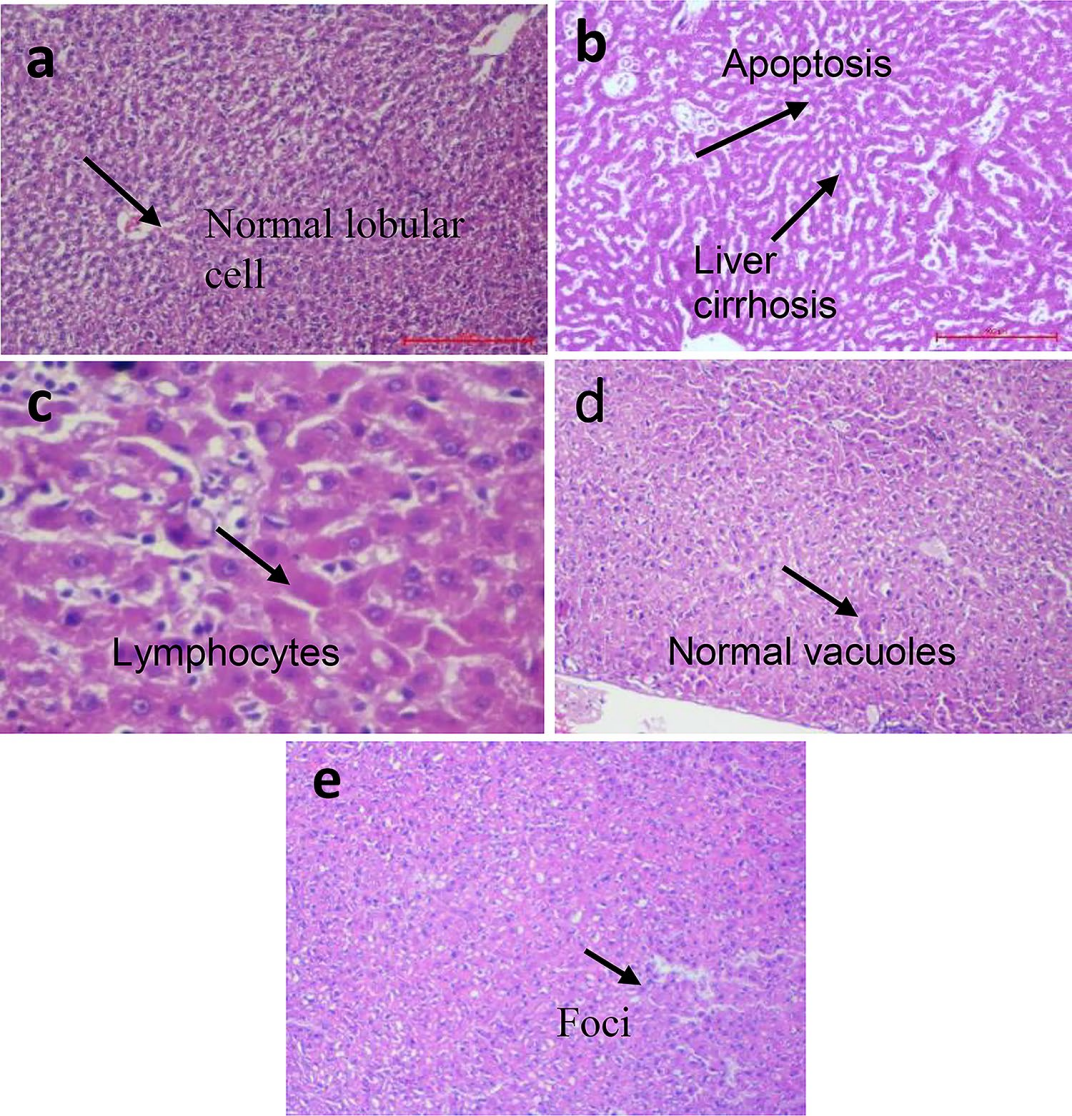
(**a**) The sinusoidal architecture was normal, and there was no fibrosis in the control group. (**b**) Infiltration of lymphocytes and macrophages, inflammation and apoptosis were observed in the toxic group. (**c**) A mild lymphocytic portal infiltrate was found in the TQ suspension group; (**d**) few lymphocytes, as well as normal vacuoles, were found in TQ-SNEDDS-treated cells; and (**e**) normal hepatocyte cells and few scattered foci were found in the silymarin group

Figure 12 shows the results of the histopathological evaluations of the normal, toxic control, TQ suspension, TQ-SNEDDS, and silymarin groups. The normal group of animals had normal liver architecture and lobular framework, without inflammatory cells or necrosis. At the same time, the toxic group’s sections showed significant changes, such as parenchymal cell injury, lymphocyte and macrophage infiltration, necrosis, and degeneration. On the other hand, the animals given TQ suspension did not show signs of inflammation or necrosis, with only minor lymphocytic infiltration. In contrast to TQ suspension, TQ-SNEDDS-treated animals exhibited more pronounced normal lobular architecture, as well as few lymphocytes and normal vacuoles. The standard treatment group, silymarin, had a nearly normal appearance of the liver parenchyma with only a few scattered foci and no evidence of inflammation or necrosis in the hepatocytes. Integrating TQ into a nanoformulation instead of pure TQ or a standard drug is a crucial and beneficial strategy for treating liver diseases.

## Conclusions

As a potential delivery system for enhancing oral bioavailability and liver health, SNEDDS loaded with TQ can be synthesized using a simple, efficient, and straightforward microemulsification method. SNEDDS prepared in this study was screened using thermodynamic stability study. Reduced particle size ( ~100 nm), high TQ solubilizing capability, and slow and sustained releasing SNEDDS with improved bioavailability were obtained. Additionally, in in vivo PCM-induced hepatotoxicity model, SNEDDS formulations significantly decreased ALP, ALT, AST, bilirubin, and albumin levels. The results of a histopathological examination of liver sections substantiated the hepatoprotective activity of TQ-loaded SNEDDS. In a nutshell, the studies ratify usefulness of SNEDDS with immense potential that may address biopharmaceutical challenges associated with TQ and other similar molecules. Further detailed studies employing TQ-loaded SNEDDS can be done at the clinical level to translate into an effective hepatoprotective formulation.

## Data Availability

All data will be made available upon request.
